# What Cuts in Federal COVID-19 Funding Could Mean for Rural Communities

**DOI:** 10.1089/heq.2022.0076

**Published:** 2022-09-01

**Authors:** Sienna Ruiz, Uzoma C. Okere, Michelle Eggers, Catina O'Leary, Beth Prusacyk, Ashley J. Housten

**Affiliations:** ^1^ Division of Public Health Sciences, Department of Surgery, Washington University School of Medicine, St. Louis, Missouri, USA.; ^2^ Health Literacy Media, St. Louis, Missouri, USA.

**Keywords:** COVID-19, rural populations, rural health disparities

## Abstract

The COVID-19 pandemic has widened the health disparities between urban and rural communities as rural populations face more limited health care capacities and worse COVID-19 outcomes than their urban counterparts. When this article was written, congress was debating continuing federal funds for free COVID-19 testing, vaccines, and treatment. In this article, we discuss the potential consequences rural communities may experience should such funding fail to be approved. Peer-reviewed literature and our research indicate these budget cuts could harm rural communities' financial distress, risk of severe disease outcomes, and trust in health care systems, making continued funding for public health resources critical for vulnerable rural communities.

## Perspective

Throughout the COVID-19 pandemic, rural communities have faced significant challenges providing and accessing adequate health care delivery and health resources to combat the spread of COVID-19. Rural hospitals have struggled to admit patients into crowded intensive care units, provide necessary treatments such as oxygen due to low supply, and find and maintain staffing to properly tend to patients.^1^ High rates of uninsured low-income patients and long distances to hospitals or health centers in rural areas have exacerbated challenges related to care delivery and treatment resources.^2,3^ Rural residents with severe cases of COVID-19 may be forced to choose between driving the long distance to the nearest hospital or treating their case at home.

They may make the multiple hour drive to the nearest hospital or health clinic only to find the emergency room is not taking more patients or is sending patients to locations even further away.^4,5^ Even before the pandemic, many rural areas from Mississippi to Idaho were dealing with crumbling health infrastructures as hospitals and other health centers closed due to financial difficulties, which left rural residents without a place to go when they were sick or injured. Such closures have only increased during the pandemic, further isolating rural communities that have been disproportionately more vulnerable to high death rates and financial distress throughout this time.^6^

At the time of writing, the federal government is debating additional COVID-19 funding that will cover testing, vaccines, and treatment.^7,8^ If such funding is not approved, patients may have to front the costs of these critical public health tools that were previously free to individuals.^9^ As we have seen since the beginning of the pandemic, new COVID-19 variants have led to new waves of cases. In anticipation of the next wave, it is critically important to ensure there is adequate coverage of tests, vaccines, and treatment.

Knowledge of one's COVID-19 status, dissemination of low-cost preventive care through vaccination, and providers' ability to administer treatments that may improve disease outcomes can give individuals and communities the tools to protect themselves and their communities. For example, individuals can use these tools to know when to isolate themselves if they are positive while local governments can plan to recommend masking if testing indicates rising cases.

Failure to subsidize these public health tools would add to the negative impacts of the federal government's shutdown of the uninsured program wherein the Health Resources and Service Administration (HRSA) discontinued reimbursements for medical providers who distribute COVID-19 testing, vaccines, and treatments for uninsured Americans.^10^ Although the HRSA website provides information on resources uninsured patients may use if they are in need of COVID-19 testing and care, the dissolution of reimbursement stands to deny uninsured people easy access to critical care during a pandemic.

Furthermore, limiting the availability of low-cost testing, vaccines, or treatments to uninsured individuals and communities at large could impede people's trust in health care organizations, which is already low throughout the country,^11^ which could in turn increase COVID-19 vaccine hesitancy or skepticism in the effectiveness of various preventive measures.

The potential impacts of the suspension of federal COVID-19 funds could be detrimental for rural communities. Medical systems that are already struggling to administer vaccines and provide necessary treatments could encounter greater barriers to care.^12,13^ For rural residents, who are more likely to be low income than their urban counterparts,^14^ paying for treatment and vaccination out of pocket could further harm families' financial well-being. Compared with urban residents, rural residents are also more likely to be older,^15^ have higher rates of comorbidities,^16,17^ and experience various vulnerabilities related to health and social factors including lack of medical insurance and difficulties paying rent, among other issues such as low educational attainment or work in high-risk environments.^18^

These factors place rural residents at greater risk of more severe COVID-19 outcomes and financial stress,^19–21^ both of which have the potential to worsen in the absence of federal funding and exacerbate disparities between rural and urban residents. Despite the difficulties experienced by rural health systems and the vulnerabilities presented by rural populations, some progress has been made in rural areas as more testing sites opened and vaccination campaigns continue.^18,22^ Discontinued federal funding could threaten this progress.

Data from our study shed light on the COVID-19–related beliefs of rural residents, many of which the federal spending cuts could undermine. We surveyed 58 participants between November 2020 and March 2021 with 27 from an urban area and 31 participants from a rural area. We found rural residents in our sample were more likely to trust in the efficacy of active measures (e.g., getting treatment from a medical provider for COVID-19 or wearing a mask) than avoidant measures (e.g., avoiding crowds or restaurants). They also expressed belief in a greater magnitude of threat posed by COVID-19 than participants in our urban sample. When asked how great of a threat they felt COVID-19 posed to their family, 58.1% of participants in our rural sample responded “A great threat” compared with 18.5% of our urban sample.

In addition, 83.9% of our rural sample found the pandemic to be a great threat to their community compared with 48.1% of our urban sample with the same response. Although our sample is small, with limited generalizability, and the surveys were distributed during an earlier phase of the pandemic, our findings mirror those of other studies. These studies have found people living in rural areas may believe in the efficacy of various public health measures and in high levels of threat for their community while being less likely to engage in preventive health behaviors.^23,24^

This paradox, wherein rural residents may believe in health measures and threats while not following preventive advice, requires more resources be dedicated to rural areas so that trusted local stakeholders can encourage rural residents to take the necessary measures for the safety of themselves and their community. Stakeholders, from nurses and physicians to local government officials, can tailor public health programs to align with rural residents' beliefs on preventive measures by emphasizing the proactive nature of testing and vaccination.

Ensuring that COVID-19 treatment will come at no cost to patients could increase trust in health care providers, who can serve as reliable sources for information on the pandemic, potentially counteracting the role misinformation may play in influencing beliefs on health measures. If the federal government ceases covering COVID-19 health resources, rural communities will be deprived of tools to increase trust in reliable sources such as health care providers or local leaders. Cessation of federal funding could push this paradox in the wrong direction, causing rural communities to act counter to their beliefs in the pandemic and increase their risk of disease contraction.

Although federal funding may have a greater impact on much needed aid to rural populations, other structural solutions for rural health should be implemented as well. Systemic problems such as understaffed and underfunded hospitals, limited providers and treatment options, and de-emphasis on primary care require systemic solutions such as expanded insurance programs, federal funding for rural hospitals, increased transportation resources, and greater recruitment for providers to serve rural areas.

Furthermore, although COVID-19 cases have declined, public health experts predict the disease will continue to spread and may take large tolls on health systems.^25^ However, it will likely be disadvantaged populations, such as those in rural areas, who will continue to bear the brunt of this disease in terms of mortality, cost, and spread,^6^ and cuts in federal funding would deny COVID-19 resources to those who need it most.

## Abbreviation Used

HRSA: Health Resources and Service Administration
